# Cell-Based Drug Delivery Systems: Innovative Drug Transporters for Targeted Therapy

**DOI:** 10.3390/ijms26178143

**Published:** 2025-08-22

**Authors:** Shynggys Sergazy, Kulzhan Berikkhanova, Alexandr Gulyayev, Zarina Shulgau, Assiya Maikenova, Ruslan Bilal, Milan Terzic, Zhaxybay Zhumadilov, Mohamad Aljofan

**Affiliations:** 1 National Laboratory Astana, Nazarbayev University, Astana 010000, Kazakhstan; kberikkhanova@nu.edu.kz (K.B.); agulyayev@nu.edu.kz (A.G.); zarina4006@mail.ru (Z.S.); maikenova98@gmail.com (A.M.); 2 School of Medicine, Nazarbayev University, Astana 010000, Kazakhstan; ruslan.bilal@nu.edu.kz (R.B.); milan.terzic@nu.edu.kz (M.T.); zzhumadilov@nu.edu.kz (Z.Z.)

**Keywords:** cell-based drug delivery, targeted transport, erythrocyte carriers, leukocyte delivery systems, platelet biomimetics

## Abstract

Significant progress has been made in developing cell-based drug delivery systems that utilize the intrinsic biological properties of various cell types—erythrocytes, leukocytes, platelets, stem cells, and even spermatozoa—to improve drug targeting, bioavailability, and biocompatibility. This review presents an integrative analysis of the latest advances in cell-based drug delivery systems, focusing on their design, pharmacokinetics, cellular interactions, and therapeutic potential. We specifically focus on hybrid microrobots and membrane-coated nanocarriers as emerging biohybrid platforms. Despite these advances, translation to the clinical phase remains constrained by persistent limitations, such as immune clearance, loss of membrane integrity during cargo loading, limited tissue penetration of carrier cells, and manufacturing challenges. Finally, we highlight future directions, including CAR-cell combinations and artificial cell engineering, that promise to expand the clinical utility of cell-based drug delivery systems in oncology, infectious diseases, and regenerative medicine.

## 1. Introduction

Recently, microspheres, hydrogels, and nanoparticles have received growing attention as drug delivery systems (DDSs). Studies have confirmed the ability of such systems to enhance drug solubility, reduce toxicity, and prolong circulation time. Compared to free drugs, these nanoformulations exhibit improved stability and excellent biocompatibility. Notably, nanoparticles have emerged as prominent drug delivery systems for controlled and sustained drug release, extending plasma half-life [1]. However, nanoparticles are readily recognized and cleared by the immune system, particularly through the reticuloendothelial system, which hinders their ability to deliver drugs effectively within the biophase [2]. Despite their potential, such “non-cellular” DDSs still face significant limitations. While clinical advances in magnetically and thermally responsive systems present an innovative DDS, overcoming barriers related to stability, bioavailability, tissue penetration, sensitivity, and active targeting remains a practical challenge [3].

The development and advancement of drug delivery technologies will foster the adoption of so-called “smart polymers” and hydrogels, particularly for systems responsive to environmental stimuli such as pH, temperature, and glucose levels, which trigger changes in specific physicochemical properties, including hydrophilicity, swelling behavior, and permeability [4]. Such innovations are expected to enhance the targeting capabilities of nanocarriers and broaden their capacity to encapsulate a diverse range of therapeutic molecules [4].

**Figure 1:**
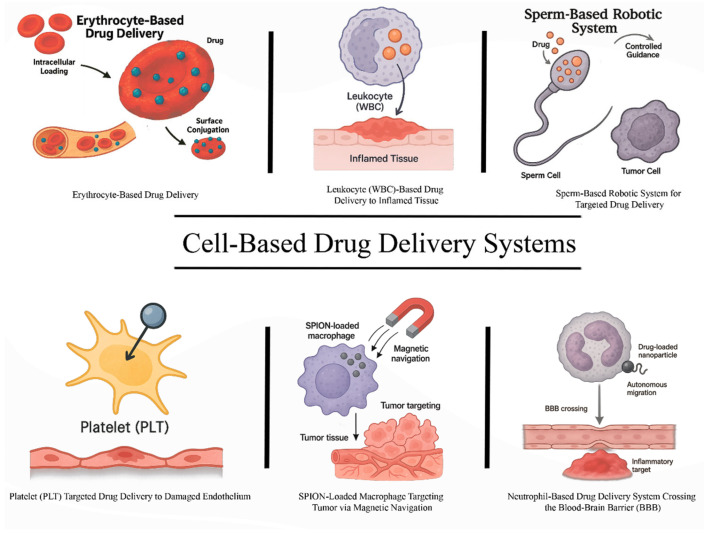
Overview of Cell-Based Drug Delivery Systems. This figure illustrates various types of cell-based drug delivery approaches. (**Top left**) Erythrocyte-based systems utilize red blood cells for either intracellular drug loading or surface conjugation to enhance circulation time and biocompatibility. (**Top center**) Leukocyte (WBC)-based delivery exploits the natural chemotactic migration of immune cells to inflamed tissue for targeted drug deposition. (**Top right**) Sperm-based robotic systems provide autonomous motility and controlled navigation toward tumor cells, facilitating localized drug delivery. (**Bottom left**) Platelets are used for targeted delivery to damaged endothelium due to their adhesion properties and natural role in vascular repair. (**Bottom center**) SPION (superparamagnetic iron oxide nanoparticles)-loaded macrophages are guided via external magnetic fields to tumor sites, combining biological targeting with physical navigation. (**Bottom right**) Neutrophils, loaded with drug-containing nanoparticles, cross the blood–brain barrier (BBB) and accumulate in inflammatory lesions, offering a promising strategy for CNS-targeted therapy.

Nevertheless, a promising strategy to overcome the inherent limitations of traditional nanomaterials lies in the development of cell-based drug delivery systems (CB-DDSs). This approach leverages the unique biological functions of cells to achieve highly controlled drug delivery, offering a level of precision that is difficult to attain with abiotic carriers. Broadly, CB-DDSs can be categorized into three main types: (1) utilization of native blood cells or their membranes to create passive carriers transported via blood flow; (2) employment of genetically or chemically modified blood cells to construct actively navigated biohybrid microrobots; and (3) application of artificial cells as a foundation for drug delivery platforms. Among the various strategies for targeted drug delivery, the use of blood components as carriers currently appears to be the most feasible. Early generations of such systems focused on native blood cells—erythrocytes, leukocytes, and platelets—aiming to establish intracellular drug depots within these cellular vehicles (Figure 1).

However, despite these obstacles, such unique properties as high immunocompatibility and the presence of natural targeting mechanisms, as well as the already demonstrated effectiveness of CB-DDSs in the treatment of severe diseases, are a compelling reason for even more in-depth study and analysis of this new method of drug delivery. Therefore, the current review outlines the primary types of existing targeted drug delivery systems, with a particular focus on those based on blood-derived cellular carriers.

## 2. Erythrocyte-Based Drug Delivery Systems

Among the cellular carriers, erythrocytes stand out due to their abundant availability, unique mechanical properties, immunosuppressive surface characteristics, and broad capacity for drug carriage [5]. Since the 1970s, erythrocytes have been extensively studied and utilized as drug carriers, taking advantage of their multifunctional ability to transport therapeutic agents within their structure [6]. Initial animal studies produced mixed results, possibly due to reduced biocompatibility of modified erythrocytes [7,8]. However, subsequent studies have shown promising outcomes in both experimental and clinical settings [9,10]. Clinical investigations have further confirmed the potential of erythrocyte-based drug carriers [11,12].

### Advantages and Strategies of Erythrocyte-Based Drug Delivery

A major and indisputable advantage of erythrocytes as drug delivery systems lies in their excellent biological compatibility [13]. It has been suggested that organ specificity in blood cells is determined by the biological behavior of these cells: leukocytes migrate to sites of inflammation, erythrocytes are sequestered by erythrophagocytic cells (mainly in the liver and spleen), and platelets adhere to damaged vascular endothelium. Current erythrocyte-based drug delivery strategies include both native erythrocytes and erythrocyte ghosts [14].

There are internal and external methodologies for drug loading into erythrocytes, including encapsulation within genetically modified erythrocytes or artificial antigen-presenting cells derived from erythrocytes [15,16]. However, the two main methods for loading drugs into erythrocytes are osmotic loading and endocytosis loading [17]. Osmotic loading involves the application of physical forces to temporarily create pores in the erythrocyte membrane. These pores are typically small, permitting the entry of molecules smaller than 50 nm, including enzymes, antigens, dexamethasone, and nanoformulations [18,19,20].

The external drug loading into erythrocytes includes chemical conjugation, cluster-specific ligand binding to surface membrane determinants, and genetic modification of reticulocytes [21].

In principle, two primary methods are used for drug loading into non-modified erythrocytes: intracellular loading and surface conjugation. Since the earliest experimental efforts to use erythrocytes for drug delivery, most researchers have focused on encapsulating pharmacological agents within the intracellular space of red blood cells [6]. However, this process typically involves the partial loss of hemoglobin and other cytosolic proteins, which are normally confined within the erythrocyte’s aqueous interior.

By contrast, surface binding of drugs to erythrocytes avoids membrane damage caused by pore formation or transmembrane permeability required for internal encapsulation. It also bypasses the need to develop controlled-release mechanisms for encapsulated drugs—a challenge that remains largely unresolved in practical settings [12]. Additionally, surface loading can be accomplished in a single step, either in vitro or in vivo. Chemical conjugation of antigens and immunoglobulins to erythrocyte membranes is also a recognized strategy for surface drug loading [22]. However, the use of non-specific cross-linking agents such as glutaraldehyde and tannic acid was associated with uncontrolled binding sites and significant membrane alterations. These methods have been largely superseded by conjugation approaches targeting erythrocyte amino acids, sulfhydryl groups, sugars, and lipids [23]. Nonetheless, the biocompatibility of modified erythrocytes remained a persistent challenge [23].

However, the development of more precisely controlled conjugation strategies, which preserve complement inhibitors like decay-accelerating factor (DAF) and CD59 on the erythrocyte membrane, enabled the surface binding of drugs with minimal membrane disruption [24]. In preclinical models, the circulation time of drug–erythrocyte conjugates was found to be similar to that of unmodified erythrocytes for several days following injection in mice and rats.

Interestingly, ligands used for affinity-based loading onto erythrocyte surface determinants include antibodies, antibody fragments, recombinant formats such as single-chain variable fragments (scFvs), heavy-chain-only camelid nanobodies, and affinity peptides. Among IgG-derived ligands, this trajectory began with polyclonal antibodies in early studies and gradually shifted to monoclonal antibodies over the past few decades. Nevertheless, all antibody-based ligands present translational challenges and may exert unintended effects on erythrocytes, including epitope clustering, cross-linking, agglutination, rigidity, Fc-mediated phagocytosis, and complement activation. Recombinant ligands like scFvs and nanobodies, which lack Fc domains and are monovalent, help mitigate these risks [25].

The erythrocyte ghost-loading method has become a widely used and convenient technique for creating drug depots within red blood cells. Loading into erythrocyte ghosts allows for prolonged release and improved pharmacokinetics of the encapsulated agent [26,27]. These carriers facilitate enhanced circulation time of therapeutic agents [28]. A wide range of biologics (e.g., antibiotics, enzymes, antigens), chemical compounds (e.g., dexamethasone), contrast agents, and other molecules have been successfully loaded into erythrocytes [29,30]. Despite their advantageous properties, the micron-scale size of erythrocytes limits their ability to diffuse beyond the vasculature and directly interact with bacterial or tumor cells, particularly in the context of treating infections or solid tumors. This limitation hinders the ability to achieve precise ligand–receptor-mediated drug targeting to tumor cells. However, the use of magnetic carriers has shown promise in overcoming these barriers by applying external magnetic fields to enhance therapeutic agent accumulation at the tumor site [31].

Guo et al. (2020) demonstrated that erythrocyte-based microrobots preserved key native features of red blood cells, including oxygen-carrying capacity, shape, deformability, and extended circulation time, highlighting their potential for advanced biomedical applications [32]. Additionally, the development of multifunctional RBC-based robots encapsulating quantum dots, the anticancer drug Adriamycin, and magnetic nanoparticles represent a major leap in theranostic technology [33]. These advances allow for simultaneous therapy and diagnostics, showcasing the versatility and translational potential of microrobots in clinical settings.

Moreover, using RBC-mimetic micromotors for oxygen delivery via hemoglobin encapsulation has shown promise in enhancing photodynamic cancer therapy [34]. The integration of RBC-based microrobots with imaging probes and therapeutic payloads forms a flexible platform capable of addressing both diagnostic and therapeutic needs. Another emerging approach, known as RBC-hitchhiking (RH), utilizes erythrocytes to transport nanoscale carriers (e.g., liposomes, nanoparticles, vesicles). In preclinical animal studies, RH has been shown to significantly increase drug accumulation in target organs [35,36]. Studies have demonstrated that RH can safely deliver drugs to selected organs via catheter-based placement in animal models and human lungs ex vivo, without inducing toxicity. For example, intra-arterial administration via the carotid artery enabled delivery of 10% of the injected dose to the brain, a tenfold improvement over traditional antibody-guided strategies [37].

While membrane-coated nanocarriers show strong clinical promise, membrane protein integrity remains difficult to preserve during extraction [38]. RH pharmacokinetics resemble those of drug-loaded erythrocyte ghosts, with preferential distribution to the liver and spleen, prolonged half-life, and reduced clearance. However, RH also dramatically alters nanoparticle behavior in the lungs: peak pulmonary uptake occurs one hour post-injection and remained high for 10 h before declining over 24 h [39]. This is attributed to pulmonary capillary architecture and hemodynamic factors promoting nanoparticle displacement from RBC surfaces—a phenomenon consistent with first-pass targeting observed in other endothelial-targeted DDSs [40]. The drug loading method via erythrocyte ghosts, extensively described by Zhumadilov and Makarenkova [41], laid the foundation for coating therapeutic agents with erythrocyte membranes, now used in designing biomimetic biohybrid micro/nanorobots [42]. One such design involved combining gold nanowires with erythrocyte-derived vesicles to create acoustically propelled micromotors.

Furthermore, magnetically guided aggregation of RBC-based carriers presents a viable approach to improve tumor accumulation of therapeutic agents [43]. These findings underscore the promise of membrane vesicle-based delivery systems and emphasize the potential of RBC-membrane cloaking strategies to advance the field of targeted drug delivery. The multifaceted applications of cell-membrane-coated micro/nanorobots, particularly those derived from erythrocytes, demonstrate significant potential for revolutionizing drug targeting, especially in cancer therapy. Cell-based micro–nanorobotic systems are now recognized as effective and biocompatible solutions for site-specific delivery [44]. Their high compatibility with biological environments can be leveraged to evade phagocytosis, reduce renal clearance, and minimize off-target toxicity [45]. The use of erythrocyte ghosts co-loaded with drugs and iron oxide nanoparticles under magnetic guidance enables both targeted delivery and sustained release at the site of action [46]. For example, the co-encapsulation of Adriamycin, quantum dots, and magnetic nanoparticles into erythrocyte-derived ghost vesicles to engineer natural RBC-based micromotors, capable of transporting multiple therapeutic agents to tumor sites [33].

Another novel application of erythrocytes in therapy is erythrocyte-based hydrogel. This hydrogel, derived from red blood cells, is used in fields such as tissue regeneration and oncology [47]. Nanoparticles derived from erythrocyte membranes have emerged as highly promising drug delivery systems due to their intrinsic biocompatibility, biodegradability, and prolonged circulation time, making them well suited for systemic administration. These properties have led to their increasing application in the development of optimized drug formulations in various preclinical models [5].

In recent years, a limited number of red blood cell-based drug delivery systems (RBC-DDS) have progressed into clinical trials, demonstrating their potential for both therapeutic intervention and diagnostic imaging [48,49]. For instance, the systematic review by Wang et al. summarizes the application of RBC membrane-coated nanoparticles (RBC-NPs) for the targeted delivery of chemotherapeutics in oncology. Typically, erythrocytes carrying therapeutic agents are administered via intravascular injection, though they have also been investigated for oral delivery [49].

Oral administration of xenogeneic erythrocytes was found to induce antibody production, potentially leading to hyperacute rejection in xenotransplant models [50]. Studies into oral delivery of drug-loaded erythrocytes remain in early phases but show potential to modulate immune responses to xenogeneic cells [51]. Oral delivery of RBCs could offer significant advantages for patients intolerant to injections or those requiring polypharmacy. However, challenges include protection from the harsh gastric environment, including pH fluctuations and digestive enzymes, as well as first-pass hepatic metabolism and intestinal absorption barriers. Overall, RBC-based drug delivery systems represent “supercarriers” capable of transporting a wide range of therapeutic agents. The extensive body of experimental data supporting these systems positions them well for translation into clinical practice, marking a critical step forward in the evolution of personalized and targeted therapy. This approach leverages the natural biocompatibility and long circulation time of erythrocytes while enhancing site-specific drug delivery.

**Figure 2:**
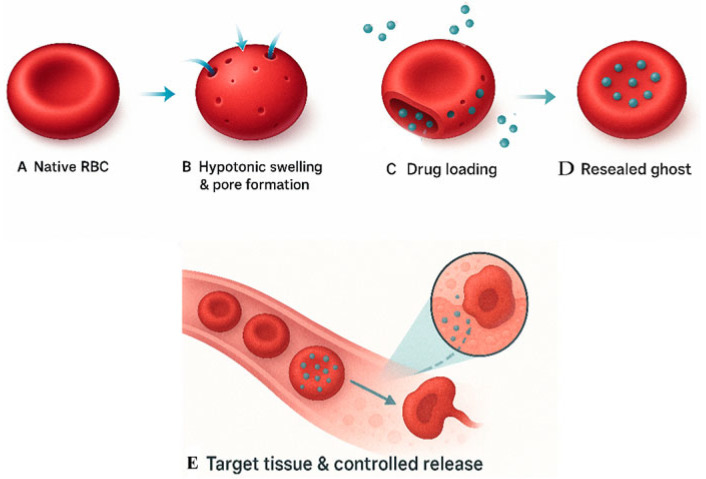
Stepwise preparation and targeted delivery mechanism of drug-loaded erythrocyte ghosts (pharmacocytes). This schematic illustration outlines the sequential stages of developing and utilizing red blood cell (RBC) ghosts for targeted drug delivery: (**A**) Native RBC: A healthy, biconcave red blood cell in its unaltered state. (**B**) Hypotonic swelling and pore formation: Exposure to a hypotonic solution causes the RBC to swell and transition toward a spherical shape, temporarily forming nanoscale pores (5–50 nm) in the membrane, depicted with blue arrows showing water influx. (**C**) Drug loading: Through these transient pores, small drug molecules (represented as teal spheres) enter the RBC’s interior. The illustration uses a cut-away view to show drug entry. (**D**) Resealed ghost: The membrane is then resealed under isotonic conditions, restoring the biconcave shape. The cell, now called a “pharmacocyte,” retains the drug payload inside its stroma. (**E**) Target tissue and controlled release: Upon intravenous infusion, pharmacocytes circulate alongside native RBCs. The delivery is directed to inflamed tissue, where interaction with immune cells (e.g., macrophages) or local pH triggers drug release. A zoomed-in inset highlights drug diffusion into the inflamed tissue.

**Figure 3:**
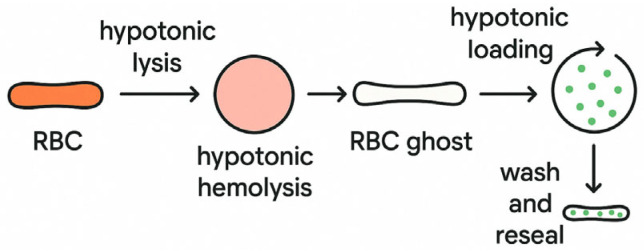
Schematic representation of the drug loading process into red blood cell (RBC) ghosts via hypotonic methods.

For drug loading into RBCs, the native RBCs undergo hypotonic lysis, resulting in the formation of permeabilized cells (ghosts) (Figure 2). This process is followed by hypotonic hemolysis, which facilitates membrane disruption. The resulting RBC ghosts are then subjected to hypotonic loading in the presence of therapeutic agents (green dots), allowing for passive diffusion of the drug molecules into the intracellular space. The final step involves washing and resealing, restoring membrane integrity to produce drug-loaded erythrocytes ready for use in targeted drug delivery applications (Figure 3).

## 3. Platelet-Based Drug Delivery Systems

Platelets have attracted considerable interest in the field of drug delivery due to their innate capacity for targeted transport. Their prolonged circulation time and ability to selectively adhere to tumor tissues, circulating tumor cells, or damaged vasculature make them ideal candidates for the targeted delivery of therapeutic agents. These characteristics position platelets as key biological components involved in various physiological and pathological processes, including hemostasis, thrombosis, immune inflammation, tumor progression, and metastasis [52].

The unique biological behavior of platelets has already led to their routine clinical use in transfusions for thrombocytopenia, as well as applications in inflammatory diseases and cancer [53]. Various loading techniques have been developed to incorporate drugs into platelets. They can be categorized into internal cargo encapsulation methods, such as hypotonic swelling and electroporation [54]. These internal encapsulation techniques allow for an increase in the concentration of the drug. However, they may negatively affect the viability and function of the platelets. For instance, electroporation can damage the cell membrane and cause irreversible membrane integrity degradation [55]. External methods of cargo loading mean binding drugs onto the cell surface. This strategy allows for protecting them from intracellular enzymes. Additionally, extracellular techniques do not compromise membrane integrity and eliminate interactions with the cell’s internal biological processes [56].

In one study, electroporated platelets loaded with a prostaglandin analogue led to a 64% reduction in platelet deposition in a rabbit atherosclerosis model [57]. Rao et al. developed a platelet-mediated photothermal therapy strategy by loading gold nanorods into platelets, achieving tumor-selective photothermal destruction in head and neck cancer models [58].

Beyond the use of live platelets, platelet membranes have been increasingly utilized for drug delivery purposes. This membrane-coating technology has undergone rapid evolution in recent decades [59]. Though platelet membranes are not intact cells, they still contain essential surface proteins such as CD47, which serve as “self” markers and help evade recognition and clearance by macrophages and the immune system [60]. Due to their exceptional targeting capabilities, platelet-mimicking drug delivery systems have found application in treating various diseases, including infection, thrombocytopenia, thrombosis, inflammation, and cancer [61,62].

In oncology, Zhang et al. developed a system by conjugating P-selectin-targeting peptide (PSN)-modified micelles to activated platelets, demonstrating effective inhibition of lung and liver metastases in triple-negative breast cancer models [63]. Another innovative strategy involves genetic engineering of platelets, offering advantages in terms of drug payload stability and production scalability. Given the anucleate nature of platelets, genetic modification must be performed on precursor cells, such as hematopoietic stem cells (HSCs), progenitors, or megakaryocytes [64]. For instance, in a murine model of multiple sclerosis, engineered platelets expressing myelin oligodendrocyte glycoprotein (MOG) successfully modulated autoimmune responses and prevented disease onset [65].

In parallel, the field is exploring the development of synthetic/artificial platelets. These biomimetic constructs aim to replicate natural platelet functions using materials such as PLGA nanoparticles functionalized with glycoprotein Ib, which allows for adhesion to damaged vascular endothelium and targeted drug delivery [66]. In one study, artificial platelets loaded with dexamethasone were able to facilitate arterial repair in a rat model [67].

Despite experimental successes, synthetic platelet systems face challenges such as low biological efficacy and high immunogenicity, limiting their current translational potential [68].

## 4. Leukocyte-Based Drug Delivery Systems

The concept of delivering antibacterial chemotherapeutics directly to infection sites was first proposed by Gulyayev et al. in 1992, who were among the pioneers in utilizing leukocytes as drug carriers [69]. The rationale was based on the ability of leukocytes to rapidly accumulate at sites of acute inflammation, making them ideal candidates for site-specific drug delivery. However, this approach presents the technical challenge of ensuring effective coupling between the drug and the cellular carrier.

It has been well established that in leukocytes—as in many other cell types—extracellular ATP can induce reversible permeabilization of the plasma membrane, allowing small, water-soluble molecules (<900 Da) to enter the cytoplasm [70,71], which can be perceived as a drawback since a lot of effective drugs have poor water solubility. This ATP-mediated permeabilization technique enables intracellular loading of drugs into leukocytes, which may be especially advantageous for cancer therapy, allowing the transport of high doses of anticancer agents using living cells while minimizing systemic toxicity.

Several strategies exist for loading leukocytes with therapeutic agents, including adsorption of drug molecules onto nanoparticles followed by cellular uptake. These particles can be phagocytosed by leukocytes and used for targeted delivery to inflamed or diseased tissues [72,73,74]. Living leukocytes offer the advantage of preserving their native membrane structures, maintaining biological activity, and enabling autonomous navigation across biological barriers [75]. In phagocytic delivery strategies, therapeutic agents are typically encapsulated into nanomaterials that are later incubated with phagocytic cells to create a drug-loaded carrier. In parallel, innovative surface modification techniques such as bacterial membrane cloaking or peptide/antibody tagging are being developed to enhance leukocyte uptake efficiency [76,77]. Moreover, unlike ATP-mediated permeabilization, the use of phagocytic uptake is not limited by molecule size and solubility, but once the nanoparticles are loaded with the drug and are within the cell, they can be susceptible to lysosomal degradation and have decreased therapeutic effect [78].

Despite these advancements, the development of living leukocyte-based DDS continues to face significant technical hurdles. Drug loading and leukocyte manipulation are predominantly performed ex vivo, which may reduce cell viability, functionality, and in vivo circulation time [79]. A considerable body of research has been dedicated to investigating the roles of different leukocyte subtypes, such as monocytes, neutrophils, and lymphocytes, in drug transport and targeting. Each subset offers distinct advantages in terms of inflammatory site homing, immune modulation, and tissue penetration, highlighting the potential for precision medicine applications using leukocyte-mediated delivery.

## 5. Neutrophils as Drug Delivery Vehicles and Components of Biohybrid Microrobots

**Figure 4:**
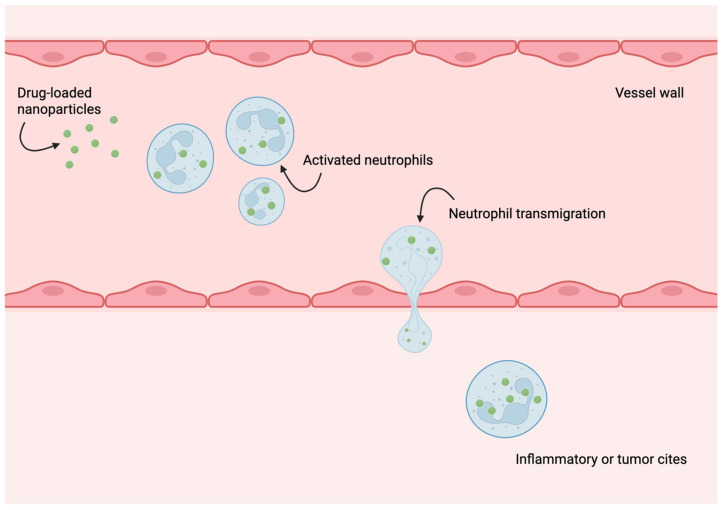
Schematic representation of neutrophil-mediated delivery of drug-loaded nanoparticles across the blood vessel barrier.

Neutrophils are among the first immune cells to migrate toward inflammatory sites during early immune responses and play a critical role in rapid disease recognition within inflamed microenvironments [80,81]. These characteristics have prompted substantial research into engineered neutrophils as universal drug delivery carriers for cancer treatment [82]. For instance, Shao et al. designed autonomous biohybrid microrobots leveraging the chemotactic ability of neutrophils, enabling self-guided migration along chemoattractant gradients to facilitate targeted drug transport [83]. Targeted delivery requires overcoming biological barriers, including the blood–brain barrier (BBB). Zhang et al. developed dual-responsive nanoelectromechanical neutrobots capable of crossing the BBB and delivering therapeutic agents directly to gliomas [84]. Another study by Chu et al. suggested that drugs or drug-loaded nanoparticles could cross the endothelial vessel wall via the neutrophil transmigration pathway (Figure 4). This hypothesis was proved by the successful delivery of the drug to lung inflammation sites [85].

These neutrophil-powered robots exhibit controlled intravascular motion under rotating magnetic fields, autonomously aggregating in brain tissues, guided by positive chemotaxis toward inflammatory gradients [86]. This represents a novel frontier for transporting drugs across otherwise restrictive barriers for treating diseases like malignant gliomas. Moreover, researchers have used leukocyte membranes, including those of neutrophils, to coat magnetic nanoparticles, enhancing circulation time, tumor cell recognition, and drug accumulation in pathological tissues [87]. Unlike erythrocyte-derived stealth coatings, neutrophil membranes offer active roles in resolving inflammation and promoting tissue regeneration [88]. Zhang et al. also engineered magnetosomes cloaked in azide-functionalized leukocyte membranes, allowing for the attachment of hydrophobic TGF-β inhibitors and PD-1 antibodies using mild click chemistry, thereby fostering an immunogenic tumor microenvironment [89]. This multifunctional approach offers precise tumor targeting and effective immunotherapy delivery, highlighting neutrophil membranes as next-generation delivery platforms.

## 6. Macrophages as Multifunctional Therapeutic Microrobots and Carriers

Macrophages, another critical class of immune cells, are characterized by exceptional phagocytic capacity, making them particularly suited for loading and transporting synthetic superparamagnetic iron oxide nanoparticles (SPIONs) while retaining viability [90]. Li et al. designed magnetically controlled immunogenic macrophage microrobots that achieve targeted multimodal cancer therapy, using magnetic manipulation and outer membrane vesicle engineering to navigate biological systems with high precision [91]. These robotic platforms show remarkable flexibility, allowing navigation through complex tissues, establishing macrophages as potent vehicles for combination cancer therapies.

Unlike erythrocytes or neutrophils, macrophages exhibit plasticity, adapting to M1 or M2 phenotypes depending on the tumor microenvironment (TME) [92]. The M1 phenotype is pro-inflammatory and tumoricidal, while M2 macrophages are often tumor-promoting [93,94]. To exploit this heterogeneity, researchers have developed macrophage membrane-coated magnetic nanorobots derived from M1-type cells, enabling photoacoustic imaging-guided photothermal immunotherapy [95]. This hybrid system enhances tumor targeting, augments immunogenicity, and improves overall therapeutic efficacy. Macrophages can be functionalized through surface conjugation or phagocytosis of drug-loaded nanoparticles, providing versatile platforms for drug transport to tumors or inflammatory foci [96]. Their self-actuating properties allow migration in response to chemotactic signals [97].

Other advantages of macrophages in drug delivery include immune evasion, BBB penetration, and high particle-loading capacity [98,99]. Moreover, activated macrophages can distinguish tumor cells from normal tissue based on differential membrane compositions, offering a “Trojan horse” mechanism for targeted delivery [100]. Recent oncopharmacological studies on macrophage-based microrobots have demonstrated promising preclinical results, underscoring their potential for clinical translation [101].

## 7. Sperm-Based Microrobots and Emerging Biohybrid Micromotors

Nature has spent millions of years perfecting flagellar propulsion, and among the microscopic cells that utilize this mode of locomotion, sperm cells stand out for their exceptional speed and efficiency [102]. Their flexible morphology allows them to navigate through narrow, tortuous pathways, and their inherent motility in viscous microenvironments makes them ideal candidates for powering microrobots [103].

**Figure 5:**
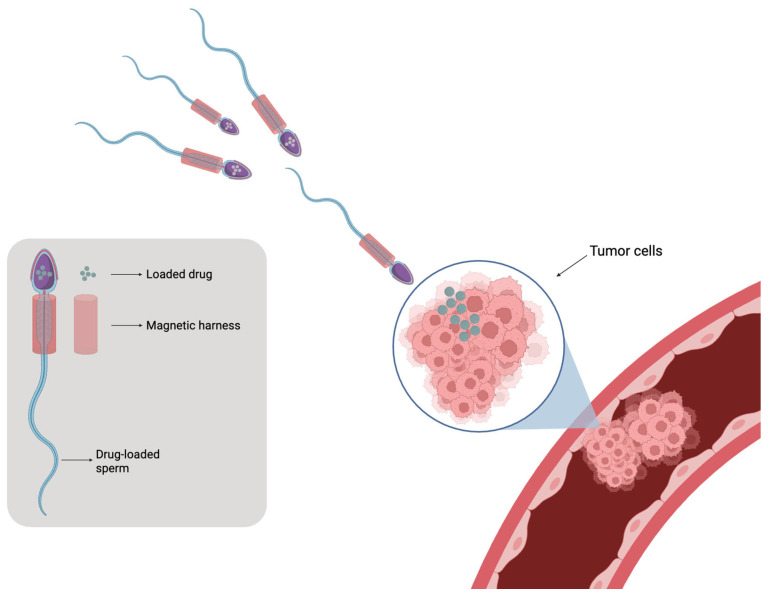
Schematic representation of sperm-based delivery of the drug to tumor tissues.

In recent years, the integration of synthetic micro/nanomaterials with sperm cells has given rise to sperm-hybrid microrobots, unlocking new biomedical applications such as assisted fertilization, gene delivery, and targeted cancer therapy [104]. Early demonstrations used bull spermatozoa due to their morphological similarity to human sperm [105]. Xu et al. demonstrated that sperm could act as biological micromotors to deliver anticancer agents (e.g., Adriamycin) directly to tumor spheroids [106]. These hybrid systems may include a 3D-printed magnetic tetrapod structure that guides non-viable sperm cells loaded with drugs, allowing for precise targeting and release, thereby minimizing accumulation in healthy tissues (Figure 5) [107].

However, the development of biohybrid microrobots—comprising living cells integrated with synthetic materials—has emerged as a transformative platform for drug delivery [108,109]. These micro/nanorobots are now employed in disease diagnostics, tumor imaging, site-specific therapy, and controlled drug delivery. Two major propulsion strategies are currently in use for these systems, including (1) self-propulsion, typically driven by catalytic reactions on robot surfaces [110], and (2) field-actuated motion, which relies on external magnetic, acoustic, optical, or electric fields for precise control [111].

Among the diverse forms of microrobots, cell-based microrobots exhibit unique biocompatibility, leveraging the innate functionality of their cellular components [112]. Flagellated bacteria were originally favored for such applications due to their autonomous motility and ease of conjugation with therapeutic nanoparticles [113]. However, limitations such as toxicity and weak actuation force restrict their widespread use [114]. By contrast, sperm-based microrobots offer naturally evolved propulsion and biocompatibility. Xu et al. proposed a hybrid system with mobile sperm cells delivering Adriamycin directly into cancer cells, establishing a new paradigm for reproductive and therapeutic microrobotics [106].

Similarly, neutrophils have emerged as potent biohybrid components. These immune cells autonomously home in on inflamed tissues via chemotaxis, engulf pathogens via phagocytosis, and have been engineered into self-guided hybrid micromotors [83,115]. Additionally, Lee et al. developed a hybrid microrobot employing click chemistry-assisted immune cell targeting, enhancing drug delivery to deep, avascular tumor regions [116]. Microrobotic technologies are rapidly advancing toward miniaturized, intelligent, and integrated systems, with efforts focusing on pairing biodegradable, biocompatible materials with living cells or microorganisms for targeted delivery. Biohybrid microrobots, when actuated by magnetic, optical, or acoustic fields, allow for remote, agile, and precise control over drug release.

Despite progress, most studies remain at the in vitro stage, with in vivo applications facing key hurdles such as precision control, circulatory navigation, and biological compatibility. The use of human-derived cells for robot fabrication is seen as a promising path forward.

There is also growing interest in artificial cells, a major goal in synthetic biology. Beyond elucidating the origins of life, artificial cells have potential as programmable drug delivery systems. Microfluidic chip technologies are now central to building artificial cells with precise structural and microenvironmental control [117,118].

Microfluidic systems allow the bottom-up assembly of synthetic cells, increasingly favored for creating advanced drug delivery platforms [119].

## 8. Useful Life of Cell-Based Drug Delivery Systems

The lifespan of CB-DDSs is a particularly important parameter that determines the therapeutic efficacy, safety, and clinical applicability. Compared to synthetic drug delivery methods, CB-DDSs have a certain lifespan in the human body, which depends on the selected cell type and varies from a few hours to several months, and sometimes even years, as in the case of macrophages [120]. For example, red blood cells, which are often used in DDSs, have a natural half-life in the human bloodstream of about 100–120 days. Such a long duration makes it possible to use red blood cells to create CB-DDSs with a slow, sustained release of drugs [121]. Neutrophil cells are very different from erythrocytes in this regard, with a half-life in the bloodstream of approximately 4 to 18 h [122], which may severely limit their use as carriers in CB-DDSs. However, they remain interesting carriers due to their ability to migrate and transport. Therefore, this feature might make them more suitable for short-term use, with a need for a rapid release of the bioactive cargo. Platelets circulate for 7–10 days; the shorter half-life of platelets compared to red blood cells or macrophages may help to obtain the required drug concentrations in a shorter time [55]. While sperm-based microrobots, as biohybrid constructs, may have a limited lifespan in vivo due to immune recognition or exhaustion of motility mechanisms, Zhang et al. reported that sperm tails did not degrade even after 10^5^ cycles in oil and aqueous environments [123].

## 9. Bioactive Molecules for Cell-Based Drug Delivery Systems and Effect of Their Physicochemical Characteristics

Numerous soluble substances that interact with and alter a cell’s activity are known as bioactive molecules. These include small-molecule drugs like doxorubicin (DOX), protein and peptides, nucleic acids, immunomodulatory agents, anti-inflammatory drugs, vaccines, and antigens. For cell-based drug delivery systems to be designed effectively, the bioactive molecule must be carefully chosen. Therefore, molecular and physicochemical characteristics of bioactive cargo have to be taken into consideration [124]. Additionally, the basic criteria for drugs and drug-loaded particles include non-toxicity to cellular carriers, minimal toxicity, biodegradability, and regulated release [125].

### 9.1. Small Molecule Drugs

Doxorubicin is one of the commonly used drugs in cancer treatment. However, its use is associated with an increased risk of heart dysfunction. For this reason, delivering the drug encapsulated within an RBC-delivery system via intravascular injection can significantly reduce toxicity while delivering the molecule to tumor tissues [126]. This assumption was confirmed in a study by Lucas et al. using a female mouse model with xenograft tumors. At the end of treatment, the group that received the RBC-DOX complex had lower concentrations of doxorubicin in the heart and skin compared to the group treated with free doxorubicin. It was also noted that mice treated with RBC-DOX had hemodynamic and cardiac function parameters closer to control values. Additionally, research teams assessed the antitumorigenic effects of RBC-DOX in human colon cancer cells (HT-29). As a result, HT-29 IC_50_ was 1.77 µg/mL for the free DOX group, while 1.45  µg/mL was the IC_50_ value for the RBC-DOX group [126]. In 2017, Xu et al. successfully incorporated doxorubicin into platelets for lymphoma treatment [127]. The designed delivery system, DOX–platelet, promoted intracellular accumulation of the drug through tumor cell-induced platelet aggregation. The results demonstrated that doxorubicin was released at low pH, indicating a pH-dependent release pattern [127]. As mentioned before, doxorubicin administration can cause cardiotoxicity and other adverse effects that limit its practical use [126]. However, the DOX–platelet system was not activated when cultured with myocardial cells, and only about 30% of DOX was released into the culture medium. Because of this, doxorubicin might not reach the threshold level needed to cause cardiotoxicity. Later results supported this hypothesis; DOX was mostly accumulated in tumor tissues in comparison with normal tissues [127].

It is also possible to combine agents and create new, more advanced cell-based drug delivery systems. For instance, the effect of doxorubicin can be significantly enhanced by encapsulating it in platelets and conjugating it with monoclonal antibodies anti-CD22 [128]. Cell viability assay showed a large difference in IC_50_ values for DOX (0.242 μg/mL), DOX–platelet (0.192 μg/mL), and DOX–platelet–CD22 (0.09 μg/mL). Further experiments on tumor (Raji, Mino, Jurkat) and normal immune cells (PBMC) showed that DOX–platelet–CD22 had a more pronounced cytotoxic effect on tumor cells and a reduced effect on PBMC [128].

Paclitaxel (PTX) is another important anticancer agent. Its ability to stabilize microtubules interferes with mitotic progression in cancer cells [129]. However, its clinical use is significantly limited by poor water solubility and systemic toxicity. Xue et al. attempted to overcome these issues by designing a cell-mediated drug-delivery system utilizing neutrophils carrying liposomes [130]. This neutrophil-mediated system (PTX-CL/NEs) could spot postoperative inflammatory signals, like IL-8 and CXCL1/KC, and deliver PTX directly to glioma cells. However, their results showed that treatment with PTX-CL/NEs had superior inhibitory effects on tumor recurrence in mouse models with surgically treated glioma, and no major influence on tumor inhibition in the group with primary gliomas [130].

Another study, also aimed at investigating tumor-treatment methods, by Tanaka et al., used platelets as a carrier for sorafenib and lenvatinib [131]. The experiment used a chemically induced HCC rat model. Six groups were created: placebo, sorafenib, lenvatinib, platelets without drug, platelets with sorafenib (SOR-PLT), platelets with lenvatinib (LEN-PLT). Platelets that were used for drug loading were initially isolated from tumor-bearing rats in vitro. Animals received the drug intravenously twice a week for 10 weeks. According to the results of the trial, extensive tumor necrosis was detected only in the groups receiving SOR-PLT and LEN-PLT. Also noteworthy is that, according to the LC-MS results, the concentration of sorafenib (loaded into platelets) was significantly higher in tumor tissues and not in the surrounding tissue. Such selectivity of drug delivery was not detected when analyzing the group that received free sorafenib [131].

### 9.2. Protein, Peptides, and Nucleic Acids

Ihler et al. were one of the first to suggest enzyme-loaded red blood cells (RBSc) for the delivery of therapeutic proteins [6]. Once the enzyme is locked in, it is well protected from excretion, inactivation, or premature degradation and is ready to be used in disease treatment [132]. For instance, Magnani et al. loaded erythrocytes with acetaldehyde dehydrogenase (AcDH) to accelerate acetaldehyde metabolism in vivo and in vitro. Results have shown that after administration of an acute dose of ethanol, a group of mice treated with AcDH-loaded erythrocytes had lower concentrations of acetaldehyde in blood samples [133]. A study by Wayne et al. revealed that macrophages could transfer siRNA into cancer cells during co-culture [134]. The study also showed that the amount of siRNA transfer occurred in a dose-dependent manner, namely, the amount of siRNA loaded and the total number of macrophages used. In MDA-MB-468 human breast cancer cells, macrophages supplied with calcium integrin binding protein-1 (CIB1)-siRNA reduced tumorsphere development and CIB1 and KI67 mRNA expression. Furthermore, animal model studies showed that adoptively transferred macrophages carrying CIB1-siRNA successfully localized to orthotopic MDA-MB-468 tumors [134].

The versatility of cell-based drug delivery systems is demonstrated by these examples. They enhance the efficacy of medications at lower dosages while enabling the direct delivery of a variety of bioactive compounds to tumors or inflammation sites. Additionally, they lessen the possibility of adverse effects.

### 9.3. Physicochemical Properties

The physicochemical characteristics of bioactive molecules have a significant impact on binding capacity and interactions with cell-based drug delivery systems. Lipophilicity and molecular size have an important role in determining loading efficiency. Additionally, they affect the retention and release of the loaded drug. Hydrophobic substances such as paclitaxel are typically distributed in the lipid bilayers of cell membranes. This property enables a more prolonged release of bioactive cargo [135]. On the contrary, hydrophilic substances are typically loaded into aqueous intracellular compartments by methods such as electroporation or sonication. However, these water-soluble molecules can also be conjugated to components on the cell surface [136]. The molecular size affects the ability of the drug to passively diffuse through biological membranes [137]. The larger the size of the molecule, the more the rate of passive diffusion decreases [137]. For this reason, more active encapsulation methods may be required.

## 10. Comparative and Critical Examination of Cell-Based Drug Delivery Systems

CB-DDSs have a major advantage, namely, the ability to target sites of disease via homing mechanisms [55]. Additionally, their circulation period in the body ranges from hours to months, allowing the choice of a drug release profile, ranging from rapid to prolonged delivery. These factors compensate for the major drawback of cell-based drug delivery systems, which is their large size, resulting in limited passive diffusion and potential entrapment in small vessels, such as capillaries, postcapillary venules, or terminal arterioles [138]. Table 1 summarizes the key characteristics, therapeutic applications, and current challenges of the main CB-DDS types.

Furthermore, the disadvantage of a large cell size is alleviated by the outstanding targeting ability of these carriers. This phenomenon is explained by their ability to respond to biochemical signals produced by damaged or inflamed tissues. Once the cellular drug carrier reaches the target point in the body, the payload can be released using different methods. These methods can be passive diffusion or exocytosis [143]. Moreover, external stimuli like enzymatic activity, oxidative stress, or acidic pH can also trigger drug release from the cell. For instance, the acidic microenvironment of tumor tissues significantly affected the release of doxorubicin in the study conducted by Xu et al. [127]. Since tumor tissues exhibit abnormalities like increased stiffness, hypoxia, and high osmotic pressure [144], this kind of hostile microenvironment may cause cell death and induce direct drug release.

Summing up, each CB-DDS has its unique advantages and limitations. For instance, despite the high biocompatibility of erythrocytes and their ability to circulate in the body for a long time, enabling drugs to undergo gradual diffusion, fast leakage is still a common problem [145]. Platelets can target vascular injuries and tumor environments for the precise delivery of drugs. However, they have some serious drawbacks. Several studies have shown that platelets can lead to thrombosis [53], and in some cases, they stimulate tumor growth [146]. Among leukocytes, neutrophils and macrophages stand out for their natural ability to reach sites of inflammation and tumor areas by responding to signaling molecules such as eicosanoids, chemokines, cytokines, and adhesion molecules released by damaged tissues [147]. But unfortunately, neutrophils have a short lifespan [148], and macrophages sometimes influence immune responses in complex ways. Sperm-based microrobots are an innovative idea. Due to their morphology and active motility, spermbots can successfully reach target tissues through narrow pathways, such as the reproductive system or dense tumor tissues [103,141]. However, the standardization process is a challenge because sperm cells vary from one individual to another, resulting in different motility [149].

### Advantages of Cell-Based Drug Delivery Systems over Polymer and Liposomal Systems

Due to their natural origin, cellular DDSs have the properties of their original cells and typically demonstrate low immunogenicity [150], which significantly enhances the safety of CB-DDSs application in treating diseases, while polymeric systems can provoke immunological reactions and other toxic effects. For example, AuNPs were found to increase cell cytotoxicity, and protein-based nanoparticles exhibit hepatotoxicity and nephrotoxicity [151]. The use of liposomal systems may be hindered by the need for additional modifications, such as PEGylation, to reduce immunogenicity [152]. In addition, cell-based drug delivery systems benefit from the fact that they are metabolized and cleared from the body naturally, reducing the risk of toxic accumulation in the body. A detailed comparative analysis of these systems is presented in Table 2.

In addition, modern technologies allow for a wide range of applications by combining CB-DDSs with polymeric or liposomal delivery methods. For example, Li et al. successfully created a complex of poly(lactide-co-glycolide) nanoparticles disguised as red blood cell membranes to deliver bFGF (bFGF-RBC/NP) for the treatment of sepsis-induced cardiac injury [158]. It is also possible to encapsulate already loaded nanoparticles directly into cells, which increases the precision and effectiveness of the therapy [85]. Therefore, the systems can coexist and complement each other.

## 11. Clinical Trials and Translation of Cell-Based DDS

Although the concept of using cells as carriers for targeted drug delivery appeared in the last century, most studies remain at the preclinical stage. For example, our analysis of the ClinicalTrials.gov, WHO ICTRP, and EU Clinical Trials Register databases showed no registered trials involving sperm cells or platelets for drug delivery. However, drug encapsulation techniques using carriers such as erythrocytes, macrophages, monocytes, and neutrophils have reached the clinical level. Most research has focused on the use of red blood cells. For instance, L-asparaginase, an antitumor agent, has been loaded into erythrocytes to reduce immunological side effects. Two drug formulations, eryaspase and GRASPA, have been developed. GRASPA (NCT01518517) was used to treat patients with relapsed acute lymphoblastic leukemia. Results from the Phase III trial showed that, on average, GRASPA demonstrated better tolerability compared to free L-asparaginase. However, statistical significance was not achieved, most likely due to the small number of participants in the study groups (adult group: GRASPA = 5 patients, L-ASP = 7 patients; children group: GRASPA = 21 patients, L-ASP = 21 patients) [159]. Another clinical trial faced a similar problem. That project aimed to study the effect of dexamethasone loaded into erythrocytes to help patients with steroid-dependent Crohn’s disease (Crodex) (NCT01277289). However, the study was terminated due to difficulties in recruiting suitable participants [56].

The aforementioned eryaspase formulation is L-asparaginase encapsulated in erythrocytes. It was used in a Phase IIb clinical trial (NCT02195180) in patients with advanced pancreatic adenocarcinoma [160]. Eryaspase was administered together with a standard chemotherapy regimen, which was gemcitabine or mFOLFOX6. The final results showed a statistically significant improvement in overall survival (OS) and progression-free survival (PFS) in the overall study population. This positive outcome was expected to be confirmed in the Phase III TRYbeCA-1 trial (NCT03665441) [161]. In that study, eryaspase was also administered in combination with chemotherapy; however, the background regimens varied depending on prior treatment and included either gemcitabine/nab-paclitaxel or irinotecan/5-fluorouracil (5FU). The final results showed that the study did not reach its primary endpoint—improvement in overall survival in patients receiving eryaspase. Nevertheless, the addition of eryaspase had a positive effect on tolerability and improved survival rates in the irinotecan/5FU subgroup [161].

Drugs based on erythrocytes loaded with dexamethasone-21-phosphate have also been developed, and a Phase II trial (NCT01255358) was conducted to evaluate the effect on neurological symptoms in ataxia-telangiectasia [56]. A subsequent Phase III trial (NCT03563053) was conducted in 2018 to collect data on the efficacy and long-term safety of treatment for this disease; however, follow-up was unfortunately not performed. The efficacy of dexamethasone-21-phosphate encapsulated in erythrocytes for reducing inflammation after coronary stenting also remained inconclusive in a Phase IV clinical trial (NCT00484965) [56]. Other clinical trials testing cell-based drug delivery systems are listed in Table 3.

Most clinical research has focused on combating inflammatory processes or oncology. Therefore, it is also necessary to direct attention to the potential use of these carriers for the treatment of other diseases.

Finally, analysis of these clinical trials shows that barriers to clinical translation include small numbers of participants, difficulties in recruiting subjects, and variability in background therapy.

## 12. Discussion

Over the past three decades, significant advancements have been made in the development of cell-based drug delivery systems to enhance pharmacokinetic parameters such as bioavailability and plasma half-life and to reduce systemic toxicity of therapeutic agents. Cell-mediated delivery systems offer numerous advantages, including sustained drug release, targeted delivery, and excellent biocompatibility. The primary strategy in many modern DDSs involves encapsulating therapeutic agents within cell membranes to enhance targeting capability, reduce off-target effects, and improve overall compatibility [162]. This biomimetic encapsulation prolongs drug circulation and facilitates more efficient accumulation at pathological sites [163].

While clinical translation of these technologies faces ongoing challenges, promising preclinical outcomes with high targeting precision and reduced side effects have been achieved. Both RBCs and leukocytes have been widely tested due to their abundance and circulatory mobility, while platelets have shown efficacy in targeting tumor vasculature and metastases [145,164]. As outlined by Rong et al., the therapeutic potential of blood cell-based DDSs is multifaceted [165]. They are widely employed due to their long circulation time, which extends systemic drug exposure. Furthermore, RBC membranes can be coated onto nanoparticles for chemotherapeutic delivery, helping to maintain therapeutic concentrations within a safe range [165]. Similarly, leukocytes possess intrinsic chemotactic and phagocytic abilities, making them ideal candidates for the treatment of inflammatory diseases and tumors [165]. For instance, Chu et al. successfully alleviated lung inflammation induced by lipopolysaccharides or infection by *Pseudomonas aeruginosa* using activated neutrophils to deliver drug-loaded albumin nanoparticles directly to sites of inflammation [85].

In addition to their natural antibacterial properties, platelets serve as highly selective delivery platforms due to their unique adhesion to vascular injury sites and tumor tissues [166]. Li et al. reported that thrombin enrichment induced platelet aggregation in melanoma, which was used to deliver the anticancer cytokine interferon-gamma induced protein 10 (IP10) via formation of a platelet–IP10 complex. This complex was selectively localized to the tumor and suppressed the accumulation of FoxP3^+^ regulatory T cells [167].

Another promising cell-type DDS is mesenchymal stem cells (MSCs), but their tropism to damaged tissues and immunomodulatory capacity, through rapid clearance by the immune system, limit their potential [168]. Interestingly, cancer cells have been explored as delivery platforms for precision medicine, although they too share safety and immunogenicity concerns akin to stem cells [95].

## 13. Future Directions

Interestingly, live-cell therapies have already reached clinical use, including CAR-T (Chimeric Antigen Receptor T-cell) therapies and dendritic cell (DC) vaccines, demonstrating the feasibility and biocompatibility of cellular therapeutics in clinical settings [169,170,171]. As a next frontier, cell-mediated immunotherapies such as CAR-macrophages [172] and CAR-NK (Natural Killer) cells [173] are gaining momentum. While CAR therapies primarily aim to enhance immune responses through genetic modification, hybrid strategies combining CAR approaches with cell-based DDSs may offer highly personalized and low-toxicity treatment modalities.

Looking ahead, we anticipate that transforming natural cells into functional microrobots or engineered carriers will revolutionize drug delivery paradigms. Combining cellular precision, biomimetic compatibility, and controlled therapeutic release, cell-based delivery platforms are well-positioned to redefine therapeutic efficacy across a range of diseases. Given the current pace of development of these technologies, fusion between CAR approaches and cell carriers may occur and reach early clinical application within the next decade, particularly in the fight against cancer.

Although clinical trials using CB-DDSs have had both positive and negative results. The main reason for failure is usually the lack of safety and limitations of the technology. Also, as the analysis of data showed, there is a problem with the recruitment of suitable subjects, and small groups of patients negatively affect the statistical significance and generalizability of the results. For this reason, it is important to improve cell carriers, as well as to develop protocols that will optimize and ensure the safety of the therapies with CB-DDSs.

## Figures and Tables

**Table 1:** Comparative characteristics, applications, and challenges of the main CB-DDS types.

Type	Key Characteristics	Therapeutic Applications	Challenges	Examples of In Vivo/In Vitro Studies
Erythrocytes	Long circulation time, biocompatible	Cancer, infections, autoimmune diseases	Fast leakage of specific drugs, large size	Targeting and depletion of circulating leukocytes and cancer cells by Rituximab-loaded erythrocytes [139]
Platelets	Selectively adhere to tumor tissues, expresses CD47	Cancer, infectious diseases, gene therapy	Risk of thrombosis, tumor growth, sensitive to environment	Doxorubicin-loaded platelets enhanced the antitumor activity of DOX by regulating the expression of apoptosis-related genes and successfully reduced the growth of the lymphoma Raji cells in BALB/c nude mice [127]
Neutrophils	Rapid response toward inflammation, phagocytosis	Glioma therapy, brain inflammation	Short lifespan, fast intracellular degradation	Treatment with neutrophil-carrying liposomes that contain paclitaxel helped to significantly inhibit tumor recurrence in surgically treated glioma mouse models [130]
Macrophages	Phagocytosis, ability to cross biological barriers	Tumor immunotherapy, BBB delivery	Limited payload, interactions with non-target tissues, heterogeneity	HIV-1 suppression in mice was achieved by using bone marrow-derived macrophages loaded with indinavir-encapsulated nanoparticles [140]
Sperm Cells	Active motion, high drug encapsulation capability	Cancer, reproductive medicine	Ethical issues, motility varies between individuals, risk, accumulation in undesired tissues	Cervical cancer HeLa cells were successfully treated by doxorubicin hydrochloride encapsulated in human sperm [141]
Membrane-Coated NPs	Mimic surface properties of the parent cell, immune escape	Cancer, infection, cardiovascular disorders	Coating efficiency, targeting capability	Resveratrol nanoparticles coated with macrophage membrane effectively targeted damaged myocardial sites, improved cardiac function, and reduced infarct size in MI mice [142]

**Table 2:** Comparison of CB-DDS, liposomes, and polymer-based nanoparticles.

Parameter	CB-DDS	Liposomes	Polymer NPs
Typical sizes	Ranging from 2 to 50 μm	50 to 500 nm [153]	10 to 1000 nm [154]
Targeting mechanism	Active	Passive/active (depends on the presence of a ligand) [153]	Passive/Active (depends on the presence of a ligand) [155]
Circulation	Hours–months (depends on the type of cell: red blood cells, macrophages, etc.)	Hours–days (require PEGylation modification) [156]	Minutes–hours [157]
Toxicity	Low	Low [156]	Moderate (depends on the polymer type; immune reactions and accumulation might happen) [151]
Advantages	- Natural targeting - Short and long circulation - Low immunogenicity - Biocompatibility	- Well studied - Suitable for water- and fat-soluble preparations - Can be modified for targeting	- Control over size and shape - Can be modified in various ways
Challenges	- Large size - Complexity of storage and standardization - Some cell carriers might have adverse effects	- Quick elimination - Aggregation is possible [153]	- Toxicity risk - May cause an immune response - Stability issues [155]

**Table 3:** List of clinical trials of cell-based drug delivery systems.

Cell Type	Type of Bioactive Cargo	Application	ClinicalTrials.Gov Identifier and Phase No.	Phase No.	Status
Red blood cells	L-Asparaginase	Pancreatic cancer	NCT02195180	Phase II	Completed
			NCT03665441	Phase III	Completed
		Leukemia	NCT01518517	Phase II/III	Completed
			NCT00723346	Phase I/II	Completed
			NCT01523782	Phase II	Completed
		Leukemia (for individuals with ALL and hypersensitivity to PEG-asparaginase)	NCT03267030	Phase II	Completed
	Dexamethasone 21-phosphate	Steroid-dependent ulcerative colitis	NCT01171807	Phase II	Completed
	Dexamethasone	Steroid-dependent Crohn’s disease	NCT01277289	Phase III	Completed
	Dexamethasone sodium phosphate	Ataxia telangiectasia	NCT01255358	Phase II	Completed
			NCT03563053	N/A	Terminated by sponsor
	L-asparaginase	Leukemia	NCT01518517	Phase II/III	Completed
Macrophages	Anti-HER2 CAR-M	HER2-positive adenocarcinoma	NCT04660929	Phase I	Active, not recruiting
Monocytes	CMV pp65-LAMP mRNA	Glioblastoma	NCT04741984	Phase I	Withdrawn
Neutrophils	Albumin-bound paclitaxel	Breast cancer	NCT06496724	N/A	Recruiting

N/A—not applicable

## Data Availability

No new data were created or analyzed in this study. Data sharing is not applicable to this article.
